# Citrullination of HP1γ chromodomain affects association with chromatin

**DOI:** 10.1186/s13072-019-0265-x

**Published:** 2019-04-02

**Authors:** Meike Wiese, Andrew J. Bannister, Srinjan Basu, Wayne Boucher, Kai Wohlfahrt, Maria A. Christophorou, Michael L. Nielsen, David Klenerman, Ernest D. Laue, Tony Kouzarides

**Affiliations:** 1 The Gurdon Institute, University of Cambridge, Tennis Court Road, Cambridge, CB2 1QN UK; 2 Department of Pathology, University of Cambridge, Tennis Court Road, Cambridge, CB2 1QP UK; 3 Department of Biochemistry, University of Cambridge, 80 Tennis Court Road, Cambridge, CB2 1GA UK; 4 Wellcome-MRC Cambridge Stem Cell Institute, Cambridge, CB2 1QR UK; 5 Institute of Genetics and Molecular Medicine, Western General Hospital, University of Edinburgh, Crewe Road South, Edinburgh, EH4 2XU UK; 6 Department of Proteomics, The Novo Nordisk Foundation Center for Protein Research, Faculty of Health Sciences, University of Copenhagen, Blegdamsvej 3b, 2200 Copenhagen, Denmark; 7 Department of Chemistry, University of Cambridge, Lensfield Road, Cambridge, CB2 1EW UK; 8 Max Planck Institute for Immunobiology and Epigenetics, Stuebeweg 51, 79108 Freiburg, Germany

**Keywords:** Chromatin, Citrullination, Stem cell differentiation

## Abstract

**Background:**

Stem cell differentiation involves major chromatin reorganisation, heterochromatin formation and genomic relocalisation of structural proteins, including heterochromatin protein 1 gamma (HP1γ). As the principal reader of the repressive histone marks H3K9me2/3, HP1 plays a key role in numerous processes including heterochromatin formation and maintenance.

**Results:**

We find that HP1γ is citrullinated in mouse embryonic stem cells (mESCs) and this diminishes when cells differentiate, indicating that it is a dynamically regulated post-translational modification during stem cell differentiation. Peptidylarginine deiminase 4, a known regulator of pluripotency, citrullinates HP1γ in vitro. This requires R38 and R39 within the HP1γ chromodomain, and the catalytic activity is enhanced by trimethylated H3K9 (H3K9me3) peptides. Mutation of R38 and R39, designed to mimic citrullination, affects HP1γ binding to H3K9me3-containing peptides. Using live-cell single-particle tracking, we demonstrate that R38 and R39 are important for HP1γ binding to chromatin in vivo. Furthermore, their mutation reduces the residence time of HP1γ on chromatin in differentiating mESCs.

**Conclusion:**

Citrullination is a novel post-translational modification of the structural heterochromatin protein HP1γ in mESCs that is dynamically regulated during mESC differentiation. The citrullinated residues lie within the HP1γ chromodomain and are important for H3K9me3 binding in vitro and chromatin association in vivo.

**Electronic supplementary material:**

The online version of this article (10.1186/s13072-019-0265-x) contains supplementary material, which is available to authorized users.

## Introduction

Embryonic stem cells (ESCs) are pluripotent cells with the unique ability to self-renew and differentiate into nearly every cell type of the body. This plasticity is maintained by a distinct nuclear architecture with particular epigenetic signatures including enrichment of active chromatin marks and dynamic binding of structural chromatin proteins [1, 2].

HP1 was originally described as a dominant suppressor of position-effect variegation in *Drosophila melanogaster* [3]. The mammalian HP1 protein family consists of three members: HP1α, β and γ. As the main reader of repressive histone marks H3K9me2/3, HP1 is a key factor in heterochromatin formation and maintenance [4, 5]. However, whilst HP1α and β are mainly associated with constitutive heterochromatic regions, HP1γ is predominantly found in euchromatin [6, 7], within the transcribed regions of active genes. Here, it regulates transcriptional elongation [8–11] and co-transcriptional mRNA processing [7, 12].

HP1γ plays important roles in developmental processes and cell fate decisions [13]. For instance, depletion of HP1γ in mESCs results in endodermal and neuronal differentiation defects [14, 15]. Interestingly, HP1γ is differentially localised during mESC differentiation. In mESCs, it predominantly occupies gene bodies, whereas in neuronal precursor cells (NPCs), it is significantly enriched at the promoters of active genes [14, 16]. This indicates a direct role for HP1γ regulating transcription during differentiation. However, little is known concerning how the recruitment of HP1γ and its association with specific genomic loci is regulated.

Peptidyl citrullination (deimination) is the post-translational conversion of an arginine (R) residue within a protein to the non-encoded amino acid citrulline. This leads to loss of a positive charge and a reduction in hydrogen bonding ability. It is mediated by a vertebrate-specific family of enzymes called peptidylarginine deiminases (PADIs) which are associated with the development of different pathological conditions such as autoimmunity, cancer and neurodegenerative diseases [17]. The deiminases are widely expressed in mammalian tissues, and PADIs 1, 2 and 4 have been reported to localise to the nucleus [18–20]. Histones are the best-characterised nuclear targets of PADI enzymes, and their citrullination is directly linked to transcriptional regulation [18–22].

We previously identified a role for PADI4 during reprogramming and ground state pluripotent stem cells [22], where the deiminase regulates key pluripotency genes via a mechanism involving citrullination of H1.2. This results in its eviction from chromatin and global chromatin decompaction. Here, we report that the chromodomain of HP1γ is also citrullinated in mESCs, at R38 and R39. We show that binding of HP1γ to peptides bearing H3K9me3 enhances its citrullination by PADI4 in vitro. Furthermore, mutations in HP1γ designed to mimic the loss of charge caused by citrullination inhibit binding of HP1γ to peptides harbouring H3K9me3 and reduce its residence time on chromatin in differentiating mESCs. We also observe a reduction in the level of HP1γ citrullination upon mESC differentiation. These results support a role for citrullination as a mechanism to regulate HP1γ chromatin association during stem cell differentiation.

## Results

### HP1γ chromodomain is citrullinated in vivo

**Fig. 1:**
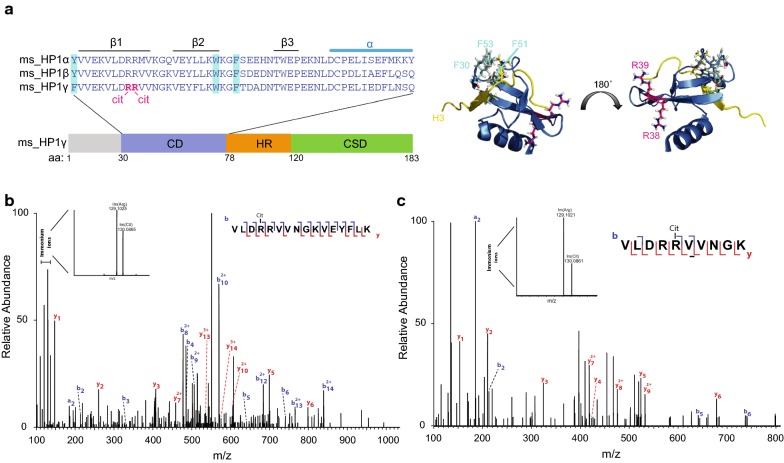
*HP1γ chromodomain is citrullinated* in vivo. **a** The left panel is a schematic illustration of the functional domain structure of mouse HP1γ. Chromodomain (CD, blue), hinge region (HR, orange), chromoshadow domain (CSD, green) and N-terminal extension (grey) are indicated. Above is a multiple sequence alignment of mouse HP1 paralogue chromodomain amino acid (aa) sequences using Clustal Omega. CD secondary structures are indicated above the sequences, and residues forming the hydrophobic H3K9me3 binding pocket [5] are highlighted in turquois. Citrullinated residues are highlighted in pink. The right panel shows an X-ray crystal structure of the HP1γ CD in complex with H3K9me3. PDB file ‘5tli’ was adapted using Pymole. Residues forming the hydrophobic pocket are highlighted in turquois and citrullinated residues in pink. **b/c** MS/MS analysis identifies citrullination of HP1γ at residues R38 and/or R39 in a chromatin fraction of mESCs. Plots show the fragmentation spectra of the LysC peptides VLDRRVVNGKVEYFLK (**b**) and VLDRRVVNGK (**c**) surrounding arginine (R) 38 and R39 of HP1γ. The y (red) and b (blue) series indicate fragments at amide bonds of the peptide. Immonium ions unique for citrulline (Im/(Cit) at *m*/*z* 130) and arginine (Im/(Arg) at *m*/*z* 129) were detected in both peptides

We previously used tandem mass spectroscopy (MS/MS) to identify citrullinated proteins in mESCs [22]. We have now performed a more thorough interrogation of these data using an improved search algorithm to identify additional citrullinated chromatin-associated proteins (Christophorou et al., manuscript in preparation). This analysis detected two citrullinated sites within the chromodomain of HP1γ (Fig. 1a, Additional file 1: Figure S1A). More specifically, the presence of immonium ions unique for citrulline (referred to as Im/(Cit) at m/z 130) and arginine (referred to as Im/(Arg) at m/z 129) was detected in two peptides (Fig. 1b, c). This strongly implies the presence of both citrulline and arginine in these peptides. In the first peptide, citrullination of R38 was identified with a localisation probability of 0.71 (Fig. 1b, Additional file 1: Figure S1B). MS/MS analysis of the second peptide identified citrullination at either R38 or R39, each with a localisation score of 0.50 (Fig. 1c, Additional file 1: Figure S1C). Taken together, our MS data indicate that HP1γ is citrullinated in mESCs at either R38 or R39, most likely the former. However, it is possible that both R38 and R39 are citrullinated.

### Efficient binding of HP1γ to H3K9me3 requires R38 and R39

**Fig. 2:**
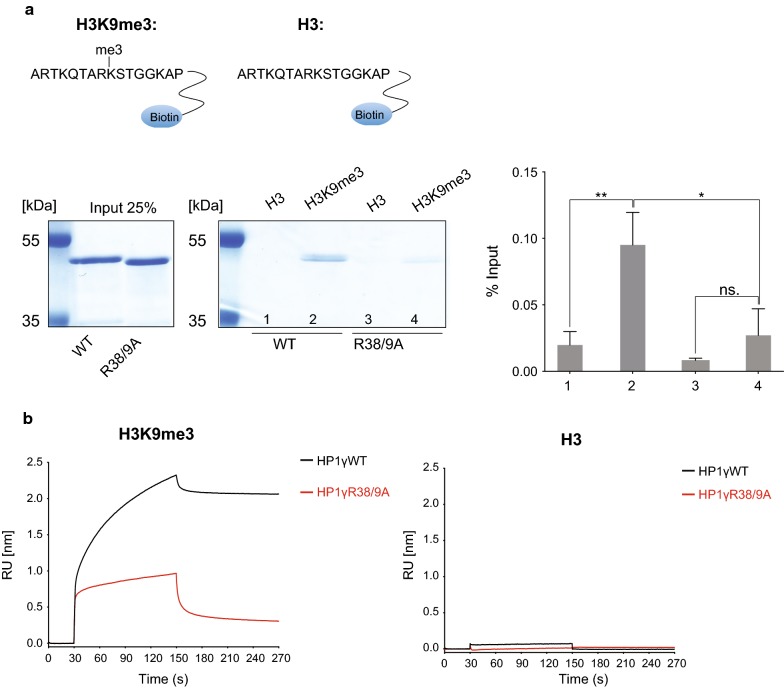
Efficient binding of HP1γ to H3K9me3 requires R38 and R39 in vitro. **a** Coomassie-blue stained gel showing the results from a pulldown assay analysing binding of recombinant GST-HP1γWT and GST-HP1γR38/9A proteins to H3(1–16) peptides or H3K9me3(1–16) peptides, as indicated. 25% of input amounts are shown. Binding intensities of gel bands from multiple experiments (Additional file 2: Figure S2A) were quantified using ImageJ and were normalised to total inputs (right hand graph). Statistical analysis was performed using unpaired, 2 tailed Student’s *t*-test. Bars represent ± SD, *n* = 3 (*p value: 0.021, **p value: 0.008, ns.: not significant, *p* value: 0.181). The sequences of the peptides used are indicated at the top. **b** Real-time binding kinetics of GST-HP1γ proteins using bio-layer interferometry (BLI). BLI sensorgrams are shown profiling the normalised binding of recombinant GST-HP1γWT and GST-HP1γR38/9A to H3(1–16) peptides (H3: right panel) and H3K9me3(1–16) peptides (H3K9me3: left panel), as indicated. Association (30–150 s) and dissociation (150–270 s) were each measured over the course of 120 s. The protein concentrations used were WT: 28.0 μM; R38/9A: 28.3 μM. Results of one experiment repeated at a range of different protein concentrations are shown (Additional file 2: Figure S2C

Citrullination of arginine neutralises the charge of the side-chain. Given that R38 and R39 are located within the chromodomain of HP1γ, we investigated whether they are important for binding H3K9me3. We generated recombinant full-length mouse HP1γ proteins, either wild type (WT) or with alanine substitutions at amino acids 38 and 39 to mimic citrullination (Additional file 1: Figure S1D). In vitro peptide pulldown assays were used to test the binding of HP1γ to unmethylated H3(1–16) or methylated H3K9me3(1–16) peptides. As expected, HP1γ specifically binds to H3K9me3 peptides (Fig. 2a, Additional file 2: Figure S2A). In contrast, we observed that binding of double alanine mutant HP1γR38/9A to H3K9me3 peptides was significantly reduced (Fig. 2a, Additional file 2: Figure S2A).

We confirmed these results by assessing real-time binding kinetics of GST-HP1γ proteins to immobilised H3 or H3K9me3 peptides using bio-layer interferometry (BLI). As expected, HP1γWT binds exclusively to H3K9me3 peptides (Fig. 2b, Additional file 2: Figure S2B and C, Additional file 3: Table S1), which agrees with previously published analyses [23]. We repeated these analyses at a range of different GST protein concentrations (Additional file 2: Figure S2C). Again, the HP1γR38/9A mutant protein displayed a distinctly different kinetic behaviour compared to the WT protein (Fig. 2b, Additional file 2: Figure S2B and C, Additional file 3: Table S1). Binding of HP1γR38/9A appears to begin to plateau at significantly lower amounts compared to the WT protein. These results confirm the data obtained from pulldown assays (Fig. 2a, Additional file 2: Figure S2A). However, a more thorough biophysical analysis is required to fully assess the kinetic behaviour of HP1γR38/9A molecules binding to H3K9me3 sites.

Interestingly, mutation of both R38 and R39 to lysine (R38/9K), which retains the positive charge at these positions, maintained binding to H3K9me3 peptides in pulldown assays (Additional file 2: Figure S2A), as well as in BLI measurements (Additional file 2: Figure S2B and C, Additional file 3: Table S1).

### PADI4 citrullinates HP1γ in vitro

**Fig. 3:**
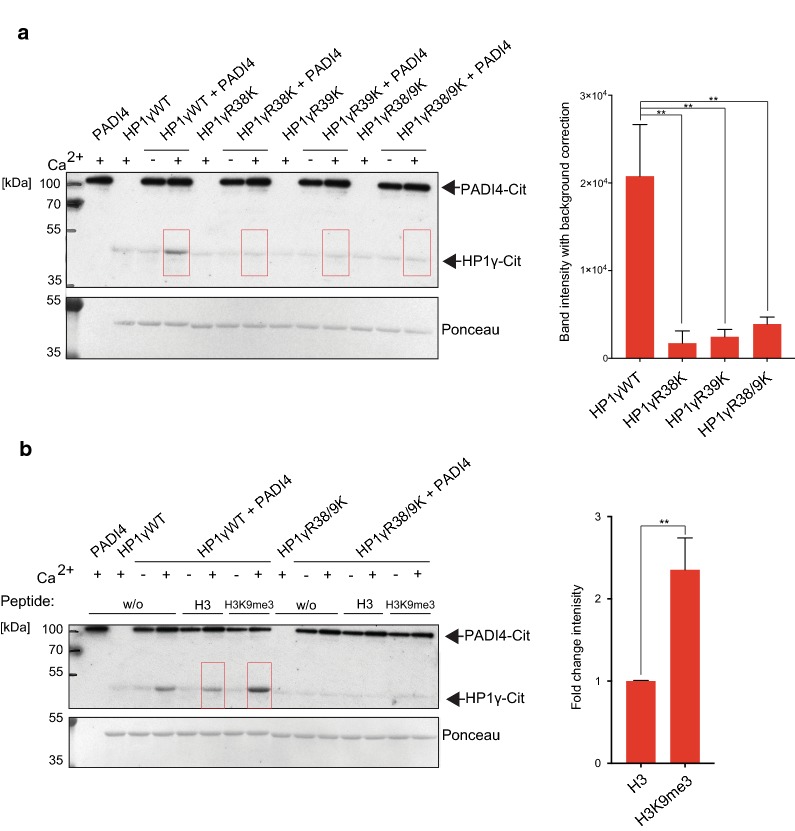
PADI4 citrullinates HP1γ in vitro.** a** Recombinant GST-HP1γWT, R38K, R39K or R38/9K mutant proteins were incubated with recombinant GST-PADI4 in the presence or absence of activating calcium as indicated. Reactions were resolved by SDS-PAGE and analysed by immunoblot analysis using an anti-peptidyl-citrulline antibody. Ponceau S staining of the transferred proteins serves as a loading control (lower panel). Bands highlighted by the red boxes were quantified using ImageJ. Total intensities after background correction are indicated (right panel). Statistical analysis was performed using two-way ANOVA multiple corrections with Sidak post hoc test. Bars represent ± SD, *n* = 3 (**p values from left to right: 0.002, 0.003, 0.008) (Additional file 4: Figure S3A). **b** GST-HP1γWT or R38/9K mutant proteins were incubated with GST-PADI4, with or without calcium, in the presence or absence (w/o) of unmethylated H3(1–16) peptides or H3K9me3(1–16) peptides, as indicated. Ponceau S staining of the transferred proteins serves as a loading control (lower panel). Bands highlighted by the red boxes were quantified using ImageJ and background corrected. Increased citrullination of HP1γWT in the presence of H3K9me3 peptides compared to unmethylated H3 peptide is displayed as fold change intensity (right panel). Statistical analysis was performed using unpaired Student’s t test. Bars represent ± SD, *n* = 3 (**p value: 0.004) (Additional file 4: Figure S3B)

PADI4 is nuclear in mESCs, and previous studies from our laboratory, as well as others, identified several chromatin-associated (histone and non-histone) substrates of the deiminase [21, 22, 24, 25]. Therefore, we next asked whether recombinant purified PADI4 citrullinates HP1γ in in vitro citrullination assays. Enzymatic activity of recombinant PADI4 was confirmed by citrullination of histone H3—a known substrate of PADI4 [18] (Additional file 4: Figure S3A and B). Using an anti-peptidyl-citrulline antibody, we found that PADI4 citrullinates HP1γ in vitro (Fig. 3a, Additional file 4: Figure S3C). To determine whether PADI4 citrullinates HP1γ at positions 38 and 39, we substituted amino acid R38 and/or R39 with lysine. Citrullination of HP1γ was abolished in both R38K and R39K mutant proteins (Fig. 3a, Additional file 4: Figure S3C), indicating that (i) both arginines are required for PADI4 to target HP1γ, and (ii) R38 and/or R39 is citrullinated by PADI4 in vitro. Specificity of HP1γ citrullination at residues R38 and R39 was further confirmed by using an affinity purified polyclonal site-specific antibody that specifically recognises HP1γ citrullination at residues R38 and R39 (Additional file 4: Figure S3D). Like the anti-peptidyl-citrulline antibody, the HP1γ-R38/9Cit antibody specifically detects citrullination of wildtype HP1γ, but not R38/9K mutant proteins in in vitro citrullination extracts (Additional file 4: Figure S3E and F), further verifying that HP1γ is citrullinated at R38 and R39 in vitro.

Interestingly, the addition of methylated H3K9me3 peptides to the in vitro citrullination assay increased HP1γ citrullination by PADI4 by approximately twofold (Fig. 3b, Additional file 4: Figure S3G). The enhanced citrullination appears specific because it was abolished when the HP1γR38/9K mutant protein was used as a substrate (Fig. 3b, Additional file 4: Figure S3G), even though this protein binds H3K9me3 peptides with similar affinity to the WT protein (Additional file 2: Figure S2). We conclude that PADI4 preferentially citrullinates HP1γ at R38 and R39 when the protein is bound to H3K9me3 peptides.

### HP1γ citrullination is reduced in differentiating mESCs

**Fig. 4:**
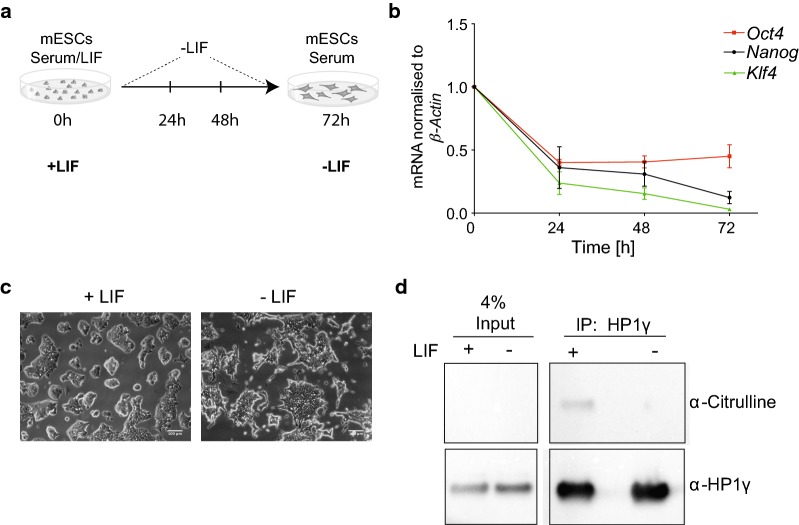
HP1γ citrullination is reduced in differentiating mESCs. **a** Schematic illustration of differentiation experiments. mESCs were cultured in the presence (+ LIF) or absence of LIF (− LIF) for 24, 48 and 72 h. Experiments were performed at 72 h. **b** mRNA levels of pluripotency markers in mESCs decrease after withdrawal of LIF. The mRNA levels of the indicated genes were measured by RT-PCR over a course of 3 days after withdrawal of LIF. RT-PCR data were normalised to *β*-*ACTIN* mRNA expression, and expression fold change was determined relative to d_0_ time point using the ddCT method. Bars represent ± SEM, *n* = 2. **c** Representative light field microscope images of mESCs before and after 72 h LIF withdrawal, captured with a Leica EC3 camera at × 20 magnification. Scale bars: 100 μm. **d** Immunoprecipitation (IP) of endogenous HP1γ from nuclear lysates of mESCs cultured +/− LIF for 72 h. IPs were performed with anti-HP1γ antibodies (α-HP1γ) and anti-HA control antibodies and analysed by immunoblotting using an anti-peptidyl-citrulline antibody (α-Citrulline). The same immunoblot was re-probed with an α-HP1γ antibody. 4% input of each IP is indicated. An uncropped image together with two more replicates is shown in Additional file 5: Figure S4A

Our data indicate that citrullination occurs in HP1γ at R38 and R39 (Figs. 1, 3). We next inquired whether citrullination of R38 and R39 could be differentially regulated in vivo. Citrullination of H1.2 by PADI4 during reprogramming of mouse embryonic fibroblasts (MEFs) highlights a role for citrullination in regulating stem cell plasticity [22]. Furthermore, both PADI4 and HP1γ are involved in regulating the integrity of pluripotent stem cells [16, 22]. We therefore assessed whether citrullination of HP1γ decreases upon stem cell differentiation, as would be expected given the decrease in *PADI4* gene expression during this process [22]. We induced differentiation in mESCs cultured under serum/LIF by LIF withdrawal for 72 h (Fig. 4a). After 72 h, the mRNA levels of the pluripotency markers *Nanog*, *Klf4* and *Oct4* were down-regulated (Fig. 4b) which was accompanied by clear morphological changes (Fig. 4c), indicating that mESCs were beginning to differentiate. We then immunoprecipitated HP1γ from nuclear lysates of mESCs cultured with or without LIF. Immunoblotting of the extracts using an anti-peptidyl-citrulline antibody showed that HP1γ is indeed significantly less citrullinated in differentiating mESCs (Fig. 4d, Additional file 5: Figure S4A). This finding not only validates the identification of HP1γ citrullination in our MS analysis (Fig. 1), but more importantly indicates that citrullination is a dynamic post-translational modification of HP1γ with a potential role in regulating stem cell plasticity.

### Mutations of HP1γ R38 and R39 do not affect soluble diffusion rate

**Fig. 5:**
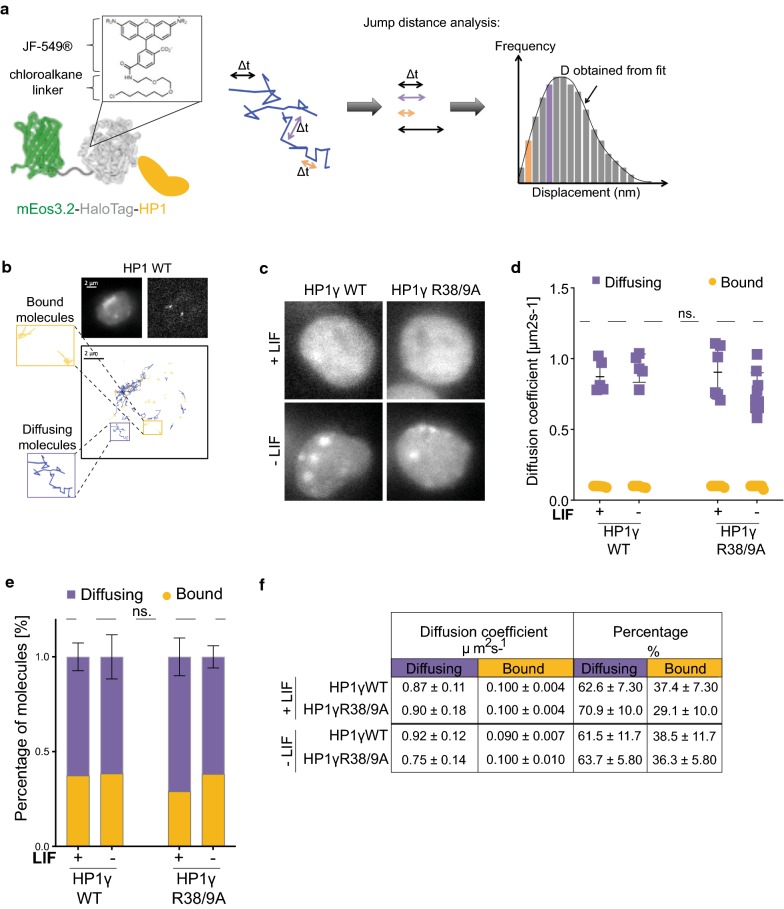
Mutation of HP1γ R38 and R39 does not affect diffusion rate of diffusing molecules. Statistical analyses were performed using two-way ANOVA multiple corrections with Sidak post hoc test. mESCs were cultured +/− LIF for 72 h. **a** Schematic illustration of mEos3.2–HaloTag–HP1 fusion proteins used for single-particle tracking (left panel). X-ray crystal structures of green EosFP (PDB_1ZUX) and HaloTag (PDB_5UXZ) are shown together with the chemical structure of JF_549_ dye coupled to the HaloTag-chloroalkane linker. Right panel shows schematic illustration of jump distance (JD) analysis of single-particle tracking (SPT) data to determine diffusion coefficients (**d**). JD analysis plots a histogram of all particle displacements within a fixed time interval Δ*t* for all trajectories. Fitting the distribution of displacements yields the minimum number of diffusion coefficients needed to describe the motion of the particles. **b** Representative mEos3.2 fluorescent images at 488 nm excitation of mESCs expressing mEos3.2–HaloTag–HP1WT and single molecule tracks of HaloTag–JF549-tagged molecules are shown. Bound and diffusing molecules are highlighted. **c** Representative mEos3.2 fluorescent images of mESCs expressing mEos3.2–HaloTag–HP1γWT and HP1γR38/9A. Scale bar: 2 μm. **d/e** SPT of mEos3.2–HaloTag–HP1γWT and HP1γR38/9A in mESCs. **d** Cells were labelled with HaloTag–JF549 ligand and subjected to 2D SPT. At 561 nm, fluorescent images were collected as movies of 10,000 frames at 13.5 ms time resolution. Plot depicts diffusion coefficients (D) [μm^2^ s^−1^] of the indicated HP1 molecules. Bars represent ± SD (*n* = 5–9). Multiple comparisons resulted in no significant differences across conditions (ns. *p* values > 0.999). **e** Percentages of molecules within diffusing and bound fractions (ns. *p* values > 0.967). **f** Tabulated summary of results shown in **d** and **e**

HP1γ R38 and R39 are important for binding to H3K9me3 peptides in vitro (Fig. 2). We then assessed whether citrullination of R38 and R39 affects association of HP1γ to chromatin in live mESCs upon LIF withdrawal. To analyse HP1γ dynamics in vivo, we used single-particle tracking of HP1γ in mESCs cultured in the presence and absence of LIF. However, although HP1 dynamics have been well characterised using numerous techniques over many years [26–29], the technique described here has not previously been used to monitor the diffusion and binding kinetics of HP1 molecules in live cells. Therefore, as a proof of principle, we tested this technique by comparing wildtype HP1γ (HP1γWT) to the well-characterised chromoshadow domain (CSD) mutant HP1γI165K, which prevents dimerisation of HP1γ [30]. We generated mESC lines stably expressing HP1N terminally tagged with the photo-activatable fluorophore mEos3.2 [31] fused to a HaloTag [32] (Fig. 5a). Expression of fusion proteins was confirmed by immunoblot analysis (Additional file 6: Figure S5A).

Tagged HP1γ molecules were labelled using low concentrations of HaloTag–JF549 ligand [33], and dynamics were measured in mESCs at 13.5 ms time resolution. Jump distance analysis as illustrated in Fig. 5a, b [34] showed that both WT and CSD mutant molecules exhibit two major diffusion coefficients (bound and diffusing) (Additional file 6: Figure S5B and C). This corresponds to published results using fluorescence correlation spectroscopy (FCS) of GFP-tagged HP1 proteins [28, 29]. Whilst diffusion coefficients of bound molecules remained unchanged for HP1γI165K mutant proteins, diffusing mutant molecules moved significantly more quickly than wildtype molecules (Additional file 6: Figure S5D to F, Additional file 7: Table S2). Overall, these results confirm previously published experiments employing fluorescence recovery after photobleaching (FRAP) of HP1 CSD mutants in both heterochromatic and euchromatic regions in vivo [26] and validate SPT as a suitable technique to detect differences in HP1 mobility.

Given the success of the above proof of principle experiments, we next compared mESCs (+/− LIF) expressing HP1γWT protein with cells expressing HP1γR38/9A, mimicking citrullinated HP1γ. Importantly, exogenous expression of HP1γ proteins had no discernible effect on mESC differentiation as (i) ground state mRNA levels of the pluripotency markers (Additional file 5: Figure S4B); (ii) down-regulation of mRNA levels of the pluripotency markers after LIF withdrawal (Additional file 5: Figure S4C); and (iii) morphological changes after LIF withdrawal (Additional file 5: Figure S4D) in mESCs stably expressing either HP1γWT or HP1γR38/9A mutant proteins did not differ from those of parental mESCs.

We observed increased HP1γ foci formation in differentiating cells by fluorescence imaging of mEos3.2 (Fig. 5c), confirming a previous study using the same differentiation protocol [35]. However, no apparent difference was detected between HP1γWT- and HP1γR38/9A-expressing cells. Similarly, SPT analyses of either HP1γWT or HP1γR38/9A mutant proteins showed that LIF withdrawal had no significant effect on their diffusion rate in mESCs (Fig. 5d–f, Additional file 7: Table S2). These results indicate that the diffusion of soluble HP1γ molecules is not affected by LIF withdrawal and it does not depend upon R38 and R39.

### HP1γ R38 and R39 determine chromatin residence time in differentiating mESCs

**Fig. 6:**
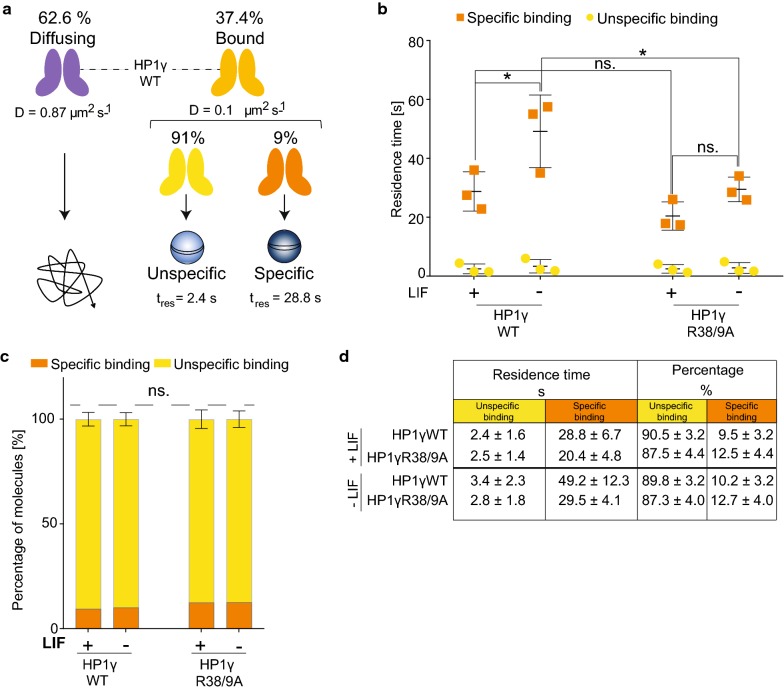
HP1γ R38 and R39 influence chromatin residence time in differentiating mESCs. Statistical analyses were performed using two-way ANOVA multiple corrections with Sidak post hoc test. mESCs were cultured +/− LIF for 72 h. **a** Schematic illustration of HP1γ dynamics in mESCs. SPT of mEos3.2–HaloTag–HP1γ molecules at 13.5 ms time resolution determines two fractions with individual diffusion coefficients: diffusing and bound. SPT at 500 ms time resolution allows further detection of molecule dynamics within the bound fraction (~ 37.4% of all detectable HP1γ molecules). Within the bound fraction, two populations with individual residence times (*t*_res_) [s] are detected: unspecific (*t*_res_ = 2.4 s) and specific (*t*_res_ = 28.8 s). **b/c** SPT of mEos3.2–HaloTag–HP1γWT and HP1γR38/9A in mESCs. **b** Cells were labelled with HaloTag–JF549 ligand and subjected to SPT. At 561 nm, fluorescent images were collected as movies of 1000 frames at 500 ms time resolution. Left panel depicts the t_res_ for specific and unspecific binding of the indicated HP1 molecules. Bars represent ± SD (*n* = 3) (*p value left to right = 0.027, 0.036; ns. *p* value > 0.841). **C** Percentages of molecules with unspecific and specific *t*_res_. **d** Tabulated summary of results shown in **b** and **c.** Errors represent ± SD (*n* = 3)

Although HP1 is a multifunctional protein, its best-characterised function is its ability to specifically bind to H3 tails harbouring H3K9me3. This underpins the establishment of localised, as well as higher-order, chromatin domains. Indeed, binding of structural chromatin proteins, such as HP1, is crucial in establishing heterochromatin during stem cell differentiation [2]. We therefore decided to focus on the chromatin bound fraction of HP1γ. We used a concept called motion blurring in which long exposures are used to blur molecules that are diffusing so they are not detected. Therefore, only molecules bound throughout the long exposure are imaged [32, 36, 37]. We performed SPT at 500 ms time resolution of mESCs expressing either HP1γWT or HP1γR38/9A mutant proteins (−/+ LIF). Movies of representative cells are shown in Additional file 8: Movie S1, Additional file 9: Movie S2, Additional file 10: Movie S3, and Additional file 11: Movie S4. Motion blurring analysis revealed that HP1 molecules bound to chromatin possess two main residence times which we will refer to as specific and unspecific residence times (Fig. 6a, Additional file 12: Figure S6, Additional file 7: Table S2).

Whilst LIF withdrawal had no effect on the unspecific residence time of either HP1γWT or HP1γR38/9A molecules, the specific residence time of HP1γWT molecules was significantly increased (20 s longer upon differentiation, Fig. 6b, d). In stark contrast, the specific residence time of R38/9A mutant HP1γ molecules was considerably shorter (Fig. 6b, d). Consequently, the average time that HP1γR38/9A molecules remain stably bound to chromatin in differentiating mESCs is significantly lower than for HP1γWT molecules (29.5 ± 4.1 vs. 49.2 ± 12.3 s (Fig. 6b, d)).

On the basis of these results, we conclude that HP1γ residues R38 and R39 are not critical for HP1 recruitment to chromatin during stem cell differentiation, because diffusion of soluble HP1γ molecules remained unchanged upon LIF withdrawal. However, the two residues influence the overall stability of HP1γ molecules bound to chromatin in differentiating mESCs.

## Discussion

**Fig. 7:**
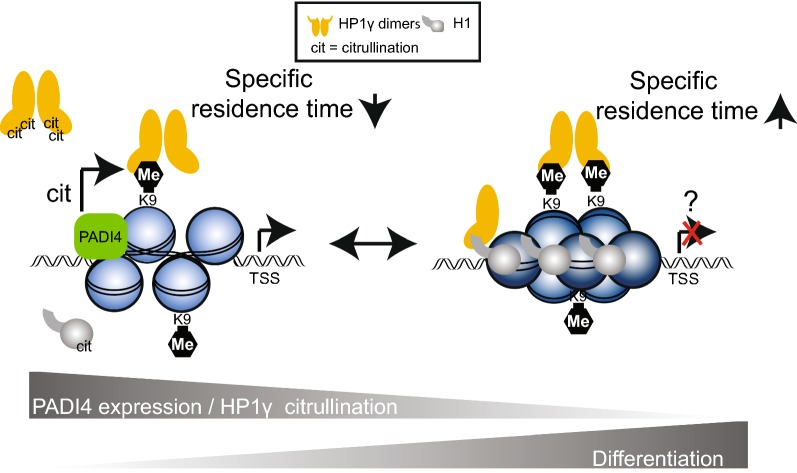
Working model depicting a role for HP1γ R38 and R39 during stem cell differentiation. In pluripotent stem cells, HP1γ proteins bound to specific genomic loci (e.g. *Nanog* promoter) are citrullinated (cit) by PADI4 to reduce their affinity to chromatin, whilst the diffusion of soluble HP1γ molecules is not affected. This may explain, at least in part, how a relatively open chromatin conformation at specific gene loci and potentially expression of pluripotency genes in stem cells is maintained. A similar (non-mutually exclusive) mechanism was identified for H1 [22], which interacts with HP1γ in stem cells with reduced pluripotent potential [39]. Upon differentiation, PADI4 expression decreases and the subsequent reduction in HP1γ citrullination would promote its interaction with chromatin (increased specific residence time) which may result in chromatin compaction and/or changes in gene expression

Our findings are consistent with a model in which HP1γ in pluripotent mESCs is citrullinated by PADI4 to reduce its affinity to chromatin (Fig. 7). This may explain, at least in part, how a relatively open chromatin conformation is maintained in pluripotent mESCs. Upon differentiation, PADI4 expression decreases [22] together with the levels of HP1γ citrullination (Fig. 4d) promoting its interaction with chromatin (increased specific residence time of HP1γ—(Fig. 6b, d)). This could help to establish heterochromatin domains and/or regulate specific gene expression programs during stem cell differentiation. Indeed, a similar non-mutually exclusive mechanism was recently reported for H1, which is also citrullinated by PADI4 [22].

Our finding that PADI4-mediated citrullination of HP1γ is significantly increased when HP1γ is bound to H3K9me3 peptides supports the notion that HP1γ is targeted by the deiminase when bound to chromatin. An X-ray crystal structure of HP1γ’s chromodomain bound to H3K9me3 (Fig. 1a) indicates that R38 and R39 are facing away from the H3K9me3 binding pocket, suggesting the residues are accessible to be targeted by an enzyme. However, to fully understand how PADI4 targeting is achieved structurally, biophysical analyses such as X-Ray crystallography or NMR of the enzyme in complex with H3K9me3 bound HP1γ will be required.

Given our in vitro citrullination data, we hypothesise that HP1γ citrullination may represent a relatively rare modification occurring at specific genomic loci to which PADI4 is recruited. Thus, HP1γ’s residence time would be affected by citrullination at specific genomic loci. In fact, our MS analysis assessed the stoichiometry of HP1γ citrullination in mESCs to be in the very low percentage range, which was supported by our immunoblotting experiments which detected relatively low levels of HP1γ citrullination in mESCs (Fig. 4d). Given this, we believe that the citrullination of HP1γ acts to regulate its chromatin binding at specific genomic sites, possibly at genes controlling cellular plasticity.

Citrullination of histone H3R8 by PADI4 at gene promoters reduces the binding affinity of HP1 to adjacent methylated H3K9 which results in gene activation [38]. It is possible that PADI4 targets both H3R8 and HP1γ bound to the same H3N-terminal tail. The *Nanog* gene may represent an important example of such a locus, since chromatin immunoprecipitation experiments have shown that both HP1γ and PADI4 bind to its promoter and regulate gene expression in stem cells [16, 22].

Additional support of our model stems from a proteomics study demonstrating HP1γ is predominantly nucleoplasmic in mESCs, with only a small fraction stably bound to chromatin [39]. In contrast, in cells with less pluripotent potential, HP1γ is more chromatin bound and predominantly interacts with H1(39), which itself is more chromatin bound [22] (Fig. 7).

HP1 proteins possess domains that allow interaction with chromatin in a multivalent fashion (reviewed in [40]). Here, we have shown that single-particle tracking can be used to reliably measure HP1 dynamics in live cells. Given the significant increase in diffusion of fast moving HP1 molecules after mutating the CSD, it is highly likely that HP1 molecules move and associate with chromatin as dimers and/or as part of larger protein complexes. The recently discovered ChAHP complex, comprised of transcription factor ADNP, chromatin remodeler CHD4 and HP1, plays a crucial role in transcriptionally regulating cell fate decisions [41]. Hence, it will be interesting to investigate how this protein complex affects HP1 dynamics in stem cells.

Finally, it is currently unclear whether citrullination of HP1γ is involved in the reprogramming of cells to a more plastic state. However, this seems plausible since loss of HP1γ increases the reprogramming efficiency of mouse embryonic fibroblasts [16].

## Conclusion

Our work identifies citrullination of HP1γ within its chromodomain as a novel post-translational modification in mESCs that is dynamically regulated during mESC differentiation. The arginines that are citrullinated within the chromodomain directly affect H3K9me3 binding in vitro and influence the overall stability of HP1γ molecules bound to chromatin in differentiating mESCs. Overall, our data highlight citrullination of HP1γ as a mechanism facilitating mESC differentiation.

## Materials and methods

### Mass spectrometry and identification of citrullinated peptides

Previously published tandem mass spectrometry data of mESCs [22] were re-analysed using an improved search algorithm. Mass spectrometry data analysis was performed with the MaxQuant software suite (version 1.2.6.20) as described [43] supported by Andromeda (www.maxquant.org) as the database search engine for peptide identifications [44]. We followed the step-by-step protocol of the MaxQuant software suite [45] to generate MS/MS peak lists that were filtered to contain at most six peaks per 100-Da interval and searched by Andromeda against a concatenated target/decoy [46] (forward and reversed) version of the Uniprot human database version (70.101 forward protein entries). Protein sequences of common contaminants such as human keratins and proteases used were added to the database. The initial mass tolerance in MS mode was set to 7 ppm, and MS/MS mass tolerance was set to 20 ppm. Cysteine carbamidomethylation was searched as a fixed modification, whereas protein *N*-acetylation, oxidised methionine, deamidation of asparagine and glutamine, and citrullination of arginines were searched as variable modifications. A maximum of two miscleavages was allowed whilst we required strict LysC specificity. Peptide assignments were statistically evaluated in a Bayesian model on the basis of sequence length and Andromeda score. We only accepted peptides and proteins with a false discovery rate of < 1%, estimated on the basis of the number of accepted reverse hits.

Modification sites within peptide sequences were assigned by calculating the localisation post-translational modification (PTM) score, which is a probability-based scoring system implemented in MaxQuant software suite and the Andromeda search engine, described previously. The score is based on the so-called MS3 scoring algorithm initially developed for the assignment of phosphorylation sites [47]. Briefly, the algorithm makes use of the 10 most intense fragment ions per 100 amu in an acquired tandem mass MS/MS spectrum. It then calculates the putative fragment ions (b- and y-ions for HCD fragmentation) in the observed mass range for all possible combinations of modification sites in a peptide sequence and determines the number of matches, *k*. The localisation PTM probability score is then derived based upon $- 10 \times {\text{log}}_{10} \left( p \right)$, where the probability *p* is calculated as

$$p = \left( {\frac{k!}{{(n!\left( {n - k} \right)!}}} \right) \times pk \times \left( {1 - p} \right)\left( {n - k} \right) = \left( {\frac{k!}{{(n!\left( {n - k} \right)!}}} \right) \times 0.04k$$

### Mammalian and bacterial expression constructs

The vector pSB7 was generated from the pEF/*myc*/ER backbone (Invitrogen) by replacing *myc*/ER regulatory elements with the sequence encoding mEos3.2–HaloTag. Mouse HP1γ cDNA was cloned into Xho1/Xba1 restriction sites of the mammalian expression vector pSB7 and into BamH1/EcoR1 restriction sites of the bacterial expression vector pGEX-2T. Point mutants were generated using Quick change II site-directed mutagenesis kit (Stratagene). Bacterial expression vector pGEX6p-GST-hPADI4 was described previously [22].

### Immunoblotting and antibodies

For immunoblotting, cells were lysed in 2 × Laemmli buffer and sonicated in a water bath (Fisherbrand) for 3 min to shear genomic DNA, spun down at full speed in a microfuge for 15 min and incubated for 5 min at 95 °C. Proteins were resolved by SDS-PAGE, transferred to nitrocellulose membrane (Millipore) using wet transfer (transfer buffer: 192 mM glycine, 25 mM Tris.HCl pH8.8) and incubated in blocking solution (5% BSA in TBS containing 0.5% Tween-20 for citrulline sensitive antibodies and 5% non-fat milk powder in TBS containing 0.1% Tween-20 for all other antibodies) for 1 h at room temperature (RT). Membranes were incubated with primary antibody at 4 °C o/n and appropriate HRP-conjugated secondary antibody for 1 h at RT. Membranes were then incubated for enhanced chemiluminescence (ECL, Promega), and proteins were detected by exposure to X-ray film or using the ChemiDoc™ MP imaging system (Bio-Rad). Images were quantified using ImageJ analysis software. Primary antibodies diluted in blocking solution were used to detect citrullinated histone H3R2 (H3R2-Cit, Abcam ab176843 at 1:1000 dilution), HP1γ (Millipore 05-690 MAB clone 42s2 [16] at 1:1000 dilution), β-Tubulin (Abcam ab6046 at 1:1000 dilution) and peptidyl-citrulline (Millipore MABN328, clone F95 [48] at 1:1000 dilution). HRP-conjugated secondary antibodies diluted in blocking solution were used against mouse IgG (Dako, P0447 at 1:5000 dilution), rabbit IgG (Abcam, ab6721 at 1:20,000 dilution) and sheep IgG (Abcam, ab6747 at 1:5000 dilution).

### Antibody purification from crude sera

Site-specific citrulline antibodies against HP1γ R38/9-citrulline were raised by Orygen Antibodies Ltd. Sheep were immunised with HP1γ peptides (aa 34–44) citrullinated at residues 38 and 39 (KVLD(Cit)(Cit)VVNGKC) that was C-terminally coupled to KLH. After the initial immunisation, 3 boost injections every 4 weeks were performed. Crude serum of the final bleed was purified against the HP1γ R38/9-citrulline peptide (same as immunogen) columns. In brief, peptides were first reduced using Bond-Breaker™ TCEP Solution (Thermofisher Scientific) and then coupled to SulfoLink™ Coupling Resin (Thermofisher Scientific) according to the manufacturer’s instructions. Non-specific binding of the column was blocked using quenching reagent (50 mM l-cysteine in coupling buffer), followed by 3 washes in wash buffer (1 M NaCl in H_2_O) and twice in 1xTBS. Crude serum was centrifuged at maximum speed for 10 min; supernatant was added to the resin and incubated at RT for 1 h on a rotating wheel. Resin was then washed 4x in 1xTBS. To elute the antibodies, elution buffer (0.2 M glycine HCl pH 2.5) was added and tube was quickly flicked and directly centrifuged at 800 rpm for 1 min. Supernatant was moved to a fresh tube and placed on ice. Immediately ice-cold neutralisation buffer (1 M Tris HCl pH 8.8) was added and mixed thoroughly to neutralise the pH to 7.

### Dot blot assay

Dot blot assays were performed by spotting synthetic peptides (HP1γ (34–44) unmodified: KVLDRRVVNGKC; HP1γR38Cit: KVLD(Cit)RVVNGKC; HP1γR39Cit: KVLDR(Cit)VVNGKC; HP1γR38/9Cit KVLD(Cit)(Cit)VVNGKC; HP1γ (104–111) R108Cit: SKKK(Cit)DAADKC; HP1α (103–112) R107Cit SKKK(Cit)EQSNDC) onto pre-wet PVDF membranes. Membranes were blocked in 5% BSA blocking buffer for 1 h at RT. Blocked membranes were then probed with the affinity purified HP1γR38/9-Cit antibody (at 1:500 dilution) at 4 °C o/n. Membranes were washed 3 times for 30 s in TBS with 0.1% Tween-20 followed by another 10 min wash in the same buffer to remove residual antibodies. Membranes were then incubated in appropriate secondary antibodies and developed as described in “Immunoblotting and antibodies” section.

### Immunoprecipitation

mESCs were either cultured under serum/LIF conditions or without LIF for 72 h. 2 × 10^7^ cells were harvested, washed twice in ice-cold 1xPBS and lysed in 1 ml of nuclear lysis buffer (NLB: 10 mM Hepes KOH pH 7.9, 1.5 mM MgCl_2_, 10 mM KCl, 0.5 mM DTT, 0.1% NP-40 supplemented with cOmplete™ proteinase inhibitor cocktail (Roche)). Lysate was incubated for 10 min on ice, and nuclei were pelleted at 2000 rpm for 10 min. Nuclear pellet was washed in NLB and lysed in 0.5 ml 1 × RIPA buffer (10 mM Tris pH 8.0, 1 mM EDTA, 1% Triton X-100, 0.1% sodium deoxycholate, 0.1% SDS, 140 mM NaCl) Supplementaled with cOmplete™ proteinase inhibitor cocktail (Roche)). The lysate was incubated on ice for 20 min with vortexing every 5 min and centrifuged at full speed for 15 min to remove cellular debris. 2 μg of HP1γ antibody (Millipore 05-690 MAB clone 42s2) or an isotype control (Abcam, HA.C5, ab18181) was added and incubated at 4 °C overnight on a rotating wheel. 30 μl of Protein G Dynabeads^®^ (Thermo Fisher Scientific) was added and incubated for 2 h at 4 °C on a rotating wheel. Beads were washed 3 ×  in ice-cold 1× RIPA, lysed in 2 × Laemmli buffer, resolved by SDS-PAGE and subjected to immunoblotting.

### Purification of recombinant GST-hPADI4 and in vitro deimination assay

Recombinant human GST-PADI4 was expressed from pGEX6p constructs [22] in LB_Amp_ media, induced with 0.1 mM IPTG at 30 °C for 4 h, purified using glutathione-Sepharose resin, eluted using a 25 mM glutathione solution and stored in 20% glycerol at − 20 °C. In vitro deimination of GST-HP1 variants was carried out as described previously [22]. In vitro deimination assays were performed in the presence and absence of 100 ng H3(1–16) or H3K9me3 peptides as appropriate. Samples were resuspended in 1 × Laemmli buffer for immunoblot analysis.

### Bio-layer interferometry (BLI)

Recombinant mouse full-length HP1 wildtype and mutant proteins were expressed in (as GST fusion proteins), and purified from, *Escherichia coli* as described [4]. Proteins were concentrated using Amicon Ultra centrifugation filters with a 30 kDa cutoff (Millipore), washed into binding buffer (20 mM Hepes KOH pH 7.2, 70 mM KCl, 1 mM DTT, 0.1% Tween-20, 5% glycerol). Protein concentrations were determined by Bradford protein assay against a BSA standard, and purity was verified by SDS-PAGE followed by Coomassie-blue staining. Real-time binding assays of GST-HP1γ were performed using the BLItz^®^ system (ForteBio) in binding buffer (+ 0.1% BSA). Biotinylated H3(1–16) peptides (NH_2_-ARTKQTARK(me3)STGGKAP-Biotin) were immobilised to Dip and Read High Precision Streptavidin (SAX) Biosensors (ForteBio), and kinetics for a range of protein concentrations were measured under advanced kinetics settings (1. initial baseline measuring buffer only: 30 s, 2. loading of peptides onto sensors: 120 s, 3. effective baseline measurement: 30 s, 4. association of GST-HP1γ proteins: 120 s, 5. dissociation of GST-HP1γ proteins: 120 s). The data were normalised to the effective baseline signal by subtracting the average baseline value (150–180 s) from each data point (150–420 s). Values of 1. and 2. measurements were not included in analysis and are not displayed in figures. Raw data are summarised in Additional file 3: Table S1.

### Peptide pulldown assay

H3K9me3 or unmethylated H3(1–16) peptides, linked to Dynabeads MyOne streptavidin T1 (Thermo Fisher Scientific), were used in pulldown assays with eluted GST-HP1 in HBS-EP (300 mM NaCl, 10 mM HEPES KOH pH 7.4, 5 mM EDTA and 0.2% NP-40). Pulldown assays were performed in 300 mM NaCl HBS-EP as previously described [4]. Samples were resuspended in 2 × Laemmli buffer and resolved by SDS-PAGE followed by Coomassie-blue staining. Images were quantified using ImageJ software.

### Cell culture

Parental E14 mouse embryonic stem cell (mESC) line was profiled by transcriptome analysis, qPCR and potency assays [22] and was routinely tested for mycoplasma contamination and tested negative. Cells were cultured in DMEM (Gibco) supplemented with 15% standard FCS (Gibco), 0.1 mM non-essential amino acids (Gibco), 1% penicillin/streptomycin, 2 mM l-glutamine (Gibco), 1 mM sodium pyruvate (Gibco), 0.1 mM β-mercaptoethanol (Life Technologies) and 10^6^ units/l mLIF (ESGRO, Millipore). E14 cells were stored in incubators at 37 °C in 7.5% CO_2_ (100% humidity) and maintained in 0.1% gelatin coated 10-cm culture dishes with 1:6 passaging every 2 days. Media was changed on a daily basis. E14 ESCs expressing mouse mEos3.2–HaloTag-HP1 fusion proteins were generated by transfecting appropriate pSB7 plasmids into E14 cells using Lipofectamine 2000 transfection reagent (Invitrogen), followed by selection in 600 µg/ml geneticin (Life Technologies). After 2 weeks of geneticin selection, cells were sorted using a Sony SH800S cell sorter for the expression of mEos3.2. Differentiation of ESCs was induced by LIF withdrawal. ESCs cultured in complete ES cell media (+ LIF) were trypsinised and washed twice in 1x PBS to remove residual LIF, seeded into ES cell media without LIF and incubated for 24, 48 or 72 h.

### cDNA synthesis and RT-PCR

Cells were lysed using QIAzol^®^ lysis reagent (Qiagen) and total RNA was isolated using RNeasy^®^ Mini Kit with in column DNase treatment (Qiagen). 1 μg of total RNA was reverse-transcribed using Superscript III Reverse Transcriptase kit (Invitrogen). Samples were diluted 1:10 in nuclease-free water prior to RT-PCR analysis. RT-PCR reactions were performed in duplicates for each sample. Each RT-PCR reaction was performed in a final volume of 10 μl. Fast SYBR Green Master Mix (Applied Biosystems) was used, according to the manufacturer’s instructions. A melting curve was obtained for each PCR product after each run, in order to confirm that the SYBR Green signal corresponded to a unique and specific amplicon. Relative expression was calculated with the 2^−ΔΔCt^ method, normalising to the housekeeping gene β-actin. Data were analysed using GraphPad Prism 7 software. Primers used in this study are summarised in Additional file 13: Table S3.

### Microscope setup

An IX73 Olympus inverted microscope was used with circularly polarised laser beams aligned and focused at the back aperture of an Olympus 1.40 NA 100 × oil objective (Universal Plan Super Apochromat, 100 × , NA 1.40, UPLSAPO100XO/1.4). Continuous wavelength diode laser light sources used include a 561-nm (Cobolt, Jive 200, 200 mW) and a 405-nm laser (Stradus, Toptica, 405–100, 100 mW). Oblique angle illumination was achieved by aligning the laser off axis such that the emergent beam at the sample interface was near-collimated and incident at an angle slightly less than the critical angle *θ*_c_ ~ 67° for a glass/water interface. This generated a ~ 50 μm diameter excitation footprint. The power of the collimated beams at the back aperture of the microscope was 10 kW/cm^2^ and 10–100 W/cm^2^ for the 561-nm and 405-nm laser beams, respectively. The lasers were reflected by dichroic mirrors (Semrock, Di01-R405/488/561/635). The fluorescence emission was collected through the same objective and then further filtered using a combination of long-pass and band-pass filters (BLP01-561R and FF01-587/35 for 561 nm excitation). The emission signal was projected onto an EMCCD (Photometrics, Evolve 512 Delta) with an electron multiplication gain of 250 ADU/photon operating in a frame transfer mode. The instrument was automated using the open-source software micromanager [49], and the data were displayed using the ImageJ software.

### Mammalian live-cell single-molecule imaging and analysis

mESCs expressing mEos3.2–HaloTag-tagged HP1γ were passaged 2 days before imaging onto 35-mm glass bottom dishes No 1.0 (MatTek Corporation P35G-1.0-14-C Case) in phenol red-free serum and LIF conditions, if not stated otherwise. Just before single-particle tracking (SPT) imaging experiments, cells were labelled with 0.5 nM HaloTag–JF549 ligand for at least 15 min, followed by two washes in PBS and a 30-min incubation at 37 °C in media, before imaging the cells in fresh phenol red-free serum and LIF conditions. Cells were under-labelled to prevent overlap of fluorophores during SPT. Using 561 nm excitation, fluorescence images were collected as movies of 10,000 frames at 13.5 ms or 1000 to 5000 frames at 500 ms exposure. For live-cell tracking, data were collected for three biological replicates on separate dishes and the average values compared. Experiments carried out on two separate days showed similar trends.

Live-cell 13.5-ms single-molecule traces were analysed using software that detects single-molecule trajectories from SPT movies [34]. Briefly, the code detected puncta positions in each image frame using a brightness-weighted centroid after applying a band-pass filter to remove low-frequency background and high-frequency noise from the image data. To track the HP1 single molecules, only fluorescent puncta smaller than 3 pixels and with a signal to noise greater than 8 were analysed. Fluorescent puncta were considered to be the same molecule if they were within 6 pixels (936 nm) between frames. We collected more than 5000 single-molecule trajectories from single-molecule movies of HP1γWT, HP1γI165K and HP1γR38/9A molecules detected within an ROI of an average of 3–4 cells. Given we observed an increase in diffusion coefficient of the fast sub-population of HP1WT molecules compared to the CSD mutants (63% of molecules for HP1γWT), this is an appropriate sample size to demonstrate the change we observe. 13.5-ms SPT experiment (i) was recorded in 1 day, and repeat experiments (*n* = 2–9, number of replicates varies between cell lines) were recorded on a different day over the course of 5 days. 500-ms data repeat experiments (*n* = 3) were recorded on three different days. Independent replicates were compared using two-way ANOVA multiple corrections with Sidak post hoc test. This is justified because the variances calculated were similar between samples.

Live-cell 500-ms single-molecule traces were analysed using Rapidstorm software that determines single-molecule localisations from PALM movies [50], after using ImageJ’s rolling back background correction with a radius of 5 pixels. Only fluorescent puncta less than 3 pixels wide with a fixed global threshold above 25,000 were analysed. We collected more than 1000 single-molecule trajectories from single-molecule movies of HP1γWT and HP1γR38/9A molecules before and after mESC differentiation. Given we observed an increase in the residence time of the stable binding HP1γWT molecules (10–13% of molecules), this is an appropriate sample size to demonstrate the change we observe.

Data were fit 1000 times after which the mean of the parameters is estimated using the parametric bootstrap model:

$$F\left( t \right) \, = \varSigma f_{i} \exp \left( { - t/\tau_{i} } \right),$$

where *f*_*i*_ is the fraction of molecules with a residence time of *τ*_*i*_. For example, a two-component fit was fit to the equation:

$$F\left( t \right) \, = \, f_{1} \exp \left( { - t/\tau_{1} } \right) \, + \, \left( {1 - f_{1} } \right)\exp \left( { - t/\tau_{2} } \right),$$

where *f*_*1*_ and (1 − *f*_1_) are the percentage of molecules with residence times of tau1 and tau2.

The code is provided on Github. We determined the relative likelihood for each model for each data set by use of the Bayesian Information Criterion (BIC) [51] as previously described for the case of comparing different diffusion models for single-particle tracking data [52]. In short, using this approach we calculated the BIC for each model from:

$${\text{BIC}} = { \ln }\left[ n \right]\left( {p + 1} \right) + n\left( {\ln \left[ {\frac{{2 \pi {\text{RSS}}}}{n}} \right] + 1} \right),$$

where *n* is the number of data points, *p* is the number of free parameters for the fit and RSS is the residual sum of squares of the fit. The fit with the lowest BIC was used (Additional file 12: Figure S6). The average values were then calculated from the fits of three independent biological replicates and compared. A *Z*-test was used to calculate the *p* values because the means are normally distributed and showed similar variance.

## Additional files

**Additional file 1: Figure S1.** Citrullination of HP1γ identified by MS/MS. **A** Multiple sequence alignment of paralogues of mouse HP1 protein sequences using Clustal Omega. Functional domains of HP1 (CD, HR and CSD) and C-terminal and N-terminal extensions are colour-coded as indicated in panel D. Isoleucine residues in the P × V × L motif, known to diminish dimerisation of HP1, are highlighted in yellow. **B/C** MS/MS fragmentation tables relating to the fragmentation spectra depicted in Fig. 1B and C, including the mass accuracies of identified fragments. **D** Schematic illustration of HP1γ mutants used in this study. Changes in amino acids are indicated. Functional domains of HP1 are illustrated: CD (blue) is the chromodomain; HR (orange) is the hinge region; and CSD (green) is the chromoshadow domain. C-terminal and N-terminal extensions are indicated in grey

**Additional file 2: Figure S2.** Mutation of R38 and R39 impairs HP1γ’s binding to H3K9me3 in vitro. **A** Unprocessed images of Coomassie-blue stained gels showing the results from pulldown assays analysing binding of GST-HP1γWT, GST-HP1γR38/9A, GST-HP1γR38/9K, GST-HP1γR38A and GST-HP1γR39A mutant proteins to H3K9me3(1–16) or unmethylated H3(1–16) peptides, as indicated. 25% of input amounts are shown. (i)–(iii) show replicates quantified in Fig. 2A. **B** BLI sensorgrams showing the normalised binding profiles of recombinant GST-HP1γWT, GST-HP1γR38/9K and GST-HP1γR38/9A binding to biotinylated H3K9me3(1–16) peptides. On the left sensorgram, association (30–150 s) and dissociation (150–270 s) were each measured over the course of 120 s. Results of one experiment are shown. Protein concentrations used are WT: 28.0 μM; R38/9K: 25.5 μM; R38/9A: 28.3 μM. **C** BLI sensorgrams showing the normalised binding profiles of recombinant GST-HP1γWT, GST-HP1γR38/9K, -HP1γR38/9A and GST binding to biotinylated H3 peptides (H3K9me3(1–16): left panel) or H3(1–16) peptides (H3: right panel). Association (30–150 s) and dissociation (150–270 s) were each measured over the course of 120 s. Results of one experiment are shown. Concentrations used from top to bottom were WT: 28.0 μM, 18.7 μM, 12.4 μM, 8.3 μM, 2.8 μM, 0.9 μM and 0.3 μM; R38/9 K: 25.5 μM, 17.0 μM, 11.3 μM, 7.6 μM, 2.5 μM, 0.8 μM and 0.3 μM; R38/9A: 28.3 μM, 18.9 μM, 12.6 μM, 8.4 μM, 2.8 μM, 0.9 μM and 0.3 μM; GST: 30.6 μM, 20.4 μM, 13.6 μM, 9.1 μM and 3.0 μM

**Additional file 3: Table S1.** Raw BLI data. Raw BLI data of GST, GST-HP1γWT, R38/9A and R38/9K proteins at different concentrations to H3K9me3(1–16) and H3(1–16) unmethylated peptides

**Additional file 4: Figure S3.** PADI4 citrullinates HP1γ in vitro. **A/B** As a known PADI4 target, recombinant H3.1 was incubated with recombinant PADI4 in the presence of activating calcium. No calcium reactions serve as negative controls. Reactions were resolved by SDS-PAGE and analysed by immunoblot analysis using (**A**) an anti-H3R2-citrulline antibody and (**B**) an anti-peptidyl-citrulline antibody (Pan-Cit). **C** Unprocessed images of in vitro citrullination assays relating to Fig. 3A. GST-HP1γWT, GST-HP1γR38K, GST-HP1γR39K or GST-HP1γR38/9K mutants were treated with GST-PADI4 in the presence or absence of activating calcium, as indicated. Reactions were resolved by SDS-PAGE and analysed by immunoblot analysis using an anti-peptidyl-citrulline antibody. Images of three biological replicates (i–iii) are shown together with their respective ImageJ quantifications. Quantifications of lanes shown in Fig. 3A are highlighted in red. D Dot blot analysis of site-specific polyclonal antibody raised against citrullinated mouse HP1γR38/9. Peptides (HP1γ(34–44) unmodified (HP1γ UM), double Cit R38/9-Cit (HP1γR38/9-Cit), single Cit R38-Cit (HP1γR38-Cit), single Cit R39-Cit (HP1γR39-Cit), single Cit R108-Cit (HP1γ(104–111)R108-Cit) and single Cit R107-Cit (HP1α(103–112)-R107-Cit)) were immobilised on PVDF membranes at indicated amounts (1–125 ng) and incubated with a purified HP1γ-R38/9-Cit antibody. **E/F** Images of in vitro citrullination assays of GST-HP1γWT or HP1γR38/9K mutant protein treated with GST-PADI4 in the presence or absence of activating calcium. Reactions were resolved by SDS-PAGE and analysed by immunoblot analysis using **(E)** a purified HP1γ-R38/9-Cit or **(F)** an anti-peptidyl-citrulline (Pan-Cit) antibody. **G** Unprocessed images of in vitro citrullination assays relating to Fig. 3B. GST-HP1γWT or -HP1γR38/9K mutant proteins were treated with GST-PADI4, with or without activating calcium, in the presence or absence of H3(1–16) or H3K9me3(1–16) peptides, as indicated. Reactions were resolved by SDS-PAGE and analysed by immunoblot analysis using an anti-peptidyl-citrulline antibody. Images of three replicates (i–iii) are shown together with their respective ImageJ quantifications. Quantifications of lanes shown in Fig. 3B are highlighted in red. Images (i–ii) depict autoradiograms whilst image (iii) was acquired using a Chemidoc™ imaging system

**Additional file 5: Figure S4.** Differentiation of mESCs. **A** Immunoprecipitation (IP) of endogenous HP1γ from nuclear lysates of mESCs before and after 72 h LIF withdrawal. IPs were performed with anti-HP1γ and anti-HA control antibodies and analysed by immunoblotting using an anti-peptidyl-citrulline antibody (α-Citrulline). Subsequently the same immunoblots were stripped and re-probed with an anti-HP1γ antibody (α-HP1γ). 4% of input amounts of each IP are indicated. Replicate (i) is shown in Fig. 4D. **B** Stable exogenous expression of mEos3.2–HaloTag–HP1γ fusion proteins does not affect total mRNA level of pluripotency markers in mESCs. RT-PCR data for the indicated genes were normalised to *β*-*actin* mRNA expression. Bars represent ± SEM (*n* = 2). **C** The mRNA levels of pluripotency markers in mESCs decrease after withdrawal of LIF. The mRNA levels of the indicated genes were measured by RT-PCR over a course of 3 days after withdrawal of LIF. RT-PCR data were normalised to *β*-*actin* mRNA expression, and expression fold change was determined relative to d_0_ time point using the ddCT method. Bars represent ± SEM (*n* = 2). **D** Representative light field microscope images of mESCs before and after 72 h LIF withdrawal, captured with a Leica EC3 camera at 20 × magnification. Scale bars: 100 μm

**Additional file 6: Figure S5.** Single-particle tracking to measure HP1 dynamics in vivo. **A** Immunoblot analysis of whole cell extracts of mESCs stably expressing or mEos3.2–HaloTag–HP1γWT or fusion proteins. Anti-HP1γ antibody detects endogenous and fusion proteins. β-Tubulin levels are shown as a loading control. **B** Representative raw SPT data of mEos3.2–HaloTag–HP1γWT and HP1γI165K molecules in mESCs. Histograms depict fraction of jumps plotted against jump distance [nm] of HaloTag–JF549-tagged HP1 in mESCs. **C** Representative jump distance (JD) analyses of mEos3.2–HaloTag–HP1γWT and I165K molecules in mESCs. Cumulative fraction of jumps plotted against JD [μm]. Yellow line indicates the result of fit to JDs of bound molecules (*D* ≤ 0.1 μm^2^ s^−1^); orange line shows the result of fit to JDs of diffusing molecules (*D* ≥ 0.6 μm^2^ s^−1^); blue line shows result of fit to JDs combining both bound and diffusing molecules; and purple line shows the raw data. **D** SPT of mEos3.2–HaloTag–HP1γWT and HP1γI165K mutant proteins. Cells were labelled with HaloTag–JF549 ligand and subjected to SPT. At 561 nm, fluorescent images were collected as movies of 10,000 frames at 13.5 ms time resolution. Plot depicts diffusion coefficients (D) [μm^2^ s^−1^] of the indicated HP1 molecules. Bars represent ± SD, *n* = 4–5, two-way ANOVA multiple comparisons with Sidak post hoc test (****p value: 0.0001). **E** Percentages of molecules within diffusing and bound fraction are shown. Bars represent ± SD (*n* = 4–5), two-way ANOVA multiple comparisons with Sidak post hoc test (ns. *p* value > 0.165). **F** Tabulated summary of results shown in **D** and **E.** Errors represent ± SD (*n* = 4–5)

**Additional file 7: Table S2.** STP data of single replicates. This file contains a summary of all biological replicates of analysed STP data of mESCs expressing HP1γWT, R38/9A or I165K mutant proteins cultured in the presence or absence of LIF for 72 h labelled with HaloTag–JF549 ligand imaged at 561 nm at 13.5 and 500 ms time resolution

**Additional file 8: Movie 1.** SPT of mESC expressing HP1γWT cultured in the presence of LIF. Movie of representative mESCs expressing HP1γWT cultured in the presence of LIF of experiment (i) labelled with HaloTag–JF549 ligand imaged at 561 nm. Fluorescent images were collected as movies of 1000 frames at 500 ms time resolution

**Additional file 9: Movie 2.** SPT of mESC expressing HP1γR38/9A cultured in the presence of LIF. Movie of representative mESCs expressing HP1γR38/9A cultured in the presence of LIF of experiment (i) labelled with HaloTag–JF549 ligand imaged at 561 nm. Fluorescent images were collected as movies of 1000 frames at 500 ms time resolution

**Additional file 10: Movie 3.** SPT of mESC expressing HP1γWT cultured in the absence of LIF. Movie of representative mESCs expressing HP1γWT cultured in the absence of LIF for 72 h of experiment (i) labelled with HaloTag–JF549 ligand imaged at 561 nm. Fluorescent images were collected as movies of 1000 frames at 500 ms time resolution

**Additional file 11: Movie 4.** SPT of mESC expressing HP1γR38/9A cultured in the absence of LIF. Movie of representative mESCs expressing HP1γR38/9A cultured in the absence of LIF for 72 h of experiment (i) labelled with HaloTag–JF549 ligand imaged at 561 nm. Fluorescent images were collected as movies of 1000 frames at 500 ms time resolution

**Additional file 12: Figure S6.** HP1γ R38 and R39 determine chromatin residence time in differentiating mESCs. Bayesian Information Criterion (BIC) of residence time modes. The relative likelihood for each model for each data set was determined by use of the BIC comparing different models for single-particle tracking data. The fit with the lowest BIC was used (light blue). Representative results for experiment (i) are shown as a proof of principle. For both HP1γWT and HP1γR38/9A, two binding modes were identified as most likely. Parameters describe: *t*_res_: residence times; *a*_1_/(*a*_1_ + *a*_2_): percentage molecules binding specifically and unspecifically

**Additional file 13: Table S3.** List of RT-PCR primers used in this study
